# Nimodipine treatment does not benefit juvenile ferrets with kaolin-induced hydrocephalus

**DOI:** 10.1186/s12987-018-0099-0

**Published:** 2018-05-03

**Authors:** Domenico L. Di Curzio, Xiaoyan Mao, Aidan Baker, Marc R. Del Bigio

**Affiliations:** 1 Department of Pathology, University of Manitoba, 401-727 McDermot Avenue, Winnipeg, MB R3E 3P5 Canada; 2 Children’s Hospital Research Institute of Manitoba, Winnipeg, MB Canada

**Keywords:** Hydrocephalus, Ferret, Kaolin, Nimodipine, Brain

## Abstract

Prior research on 3-week hydrocephalic rats showed that behavioral deficits and white matter damage could be reduced by treatment with Ca^2+^ channel blocker nimodipine. We hypothesized that treatment with nimodipine would be also beneficial to young ferrets with kaolin-induced hydrocephalus. Hydrocephalus was induced at 14 days of age and animals were treated either with vehicle, low dose nimodipine (3.2 mg/kg/day), or high dose nimodipine (16 mg/kg/day) for 2 weeks from 38 to 52 days age. Hydrocephalic ferrets developed progressive ventriculomegaly, behavioral changes, and in some cases cortical blindness. These changes were not ameliorated by nimodipine. Histological examination showed damage in periventricular white matter, corpus callosum thinning, axonal damage, reactive astroglial changes, and suppressed cell proliferation compared to non-hydrocephalic controls. Treatment with nimodipine was not beneficial for any of the pathological changes mentioned above; only low dose nimodipine treatment was associated with normalized content of glial fibrillary acidic protein, despite larger ventricles. We conclude that young hydrocephalic ferrets experience behavioral impairments and structural brain damage that are not consistently improved by intermittent nimodipine treatment. Continuous delivery should be considered in further preclinical studies.

## Introduction

Brain damage induced by hydrocephalus is multifactorial commencing with mechanical factors and local ischemia that result in destruction of periventricular axons [1]. The gradual nature of damage means that there might be an opportunity for an early pharmacologic intervention to reduce the damage prior to definitive treatment such as cerebrospinal fluid (CSF) shunting [2]. Nimodipine is a lipophilic antagonist of voltage-dependent selective dihydropyridine type (L-type) calcium (Ca^2+^) channels [3]. It can cross the blood–brain-barrier [4] and in animals has been shown to cause cerebral vasodilatation [5]. However, one small clinical study on eight adults with normal pressure hydrocephalus showed that nimodipine treatment reduced the arterial blood pressure, had no effect calculated on cerebral blood flow, and was associated with a slight increase in intracranial pressure during a 4 h monitoring period [6]. We previously showed that 3-week old rats with kaolin-induced hydrocephalus benefited behaviorally and structurally by treatment with continuous parenteral administration of nimodipine for 2 weeks [7]. In preclinical trials it is necessary to demonstrate the utility and safety of this treatment in a larger species with gyrencephalic brain [2]. Therefore, this study was aimed to determine if nimodipine would benefit juvenile ferrets [8].

## Methods

Thirty-five pigmented sable ferret kits (n = 26 males and n = 9 females) were obtained from Marshall Farms (North Rose, NY) at postnatal age 7–9 days (P7–P9) among 6 l along with their mothers. All animals were treated humanely based on the Canadian Council on Animal Care guidelines. The University of Manitoba animal ethics committee approved the experiment (protocol #15-076). The methods of animal handling, anesthesia, induction of hydrocephalus, magnetic resonance (MR) imaging, behavioral testing, euthanasia, biochemical analyses, and histological analyses were all as previously described in detail [8, 9]. Below we highlight the experimental timing, drug utilization, and any additions to prior work.

Hydrocephalus was induced in anesthetized (3.0% isoflurane) ferrets with percutaneous intracisternal injection of kaolin or sham (saline) at P14 (weight 44–81 g; 5–6 kits/l; total n = 35). Subcutaneous (sc) injections of buprenorphine (0.03 mg/kg) and sterile 0.45% saline were given every 8–12 h over 2 days to reduce pain and dehydration. T2-weighted MR images of brain were obtained using a 7 T Bruker Biospec/3 MR scanner (Karlsruhe, Germany). MR imaging at 2 days post kaolin (P16) ensured that hydrocephalus had been successfully induced; in the case of failures, an additional dose of kaolin was administered. Nine kits died after kaolin injections leaving 26 that underwent MR imaging 15 days after kaolin (at P29). Using the lateral ventricle to brain size ratios, ferrets were stratified and assigned list wise to vehicle (0 mg/kg/day), low dose nimodipine (3.2 mg/kg/day), and high dose nimodipine (16 mg/kg/day) treatment groups. The final set of MR images was obtained on P52, after 2 weeks drug therapy, no more than 24 h before sacrifice.

Stock solutions of nimodipine (Sigma, St. Louis, MO) were prepared in 40% dimethyl sulfoxide (DMSO; Fisher Scientific, Nepean ON), 10% ethanol, 50% 0.9% NaCl in distilled water and stored at 4 °C. The solutions of various nimodipine concentrations along with a sham vehicle were labeled A, B, and C. Treatments were administered blindly based upon a subcutaneous injection volume to body weight formula of 1 ml/100 g twice per day (approximately at 08.00 and 18.00 h). The nimodipine dose was either 3.2 mg/kg/day (low) or 16 mg/kg/day (high). These doses were based on the previous rodent experiment wherein nimodipine 8–13 mg/kg/day was found to be protective and 20 mg/kg/day exhibited mild toxicity [7]. Note that the doses are not entirely comparable owing to differences in administration routes (osmotic minipumps in rats). Treatments were started 15–17 days post-kaolin at P29–P31 (n = 9 vehicle; n = 9 low dose; n = 8 high dose). Treatments were temporarily halted after 2 days because several ferrets suffered lethargy and rapid weight loss. We suspected an adverse reaction to the vehicle; therefore, the drug solutions were reformulated in half the volume of vehicle. Drug administration resumed at P38, which allowed a 14-day treatment period.

Behavioral testing commenced at P9–P11 and continued three times weekly until P48–P50. Activity was measured in an enclosed square container (44 × 43 × 29 cm) with infrared light beam sensors to record vertical, ambulatory, and total movements (Opto-Varimex 3; Columbus Instruments International Corp., Columbus, OH, USA). Open field activity was video recorded in a transparent enclosure (75 × 75 × 45 cm) for quantitative assessment of ambulation. Vision was tested when the ferrets started to open their eyes (P36) using a foam ball (5 cm diameter, painted red) attached to the end of a metal stick. The ball was introduced in two different manners. First, it was held at the side of the ferret and waved (approximately 2 cm amplitude) until the animal turned its head toward the ball. Second, the ball was placed in front of the kit then moved slowly to see if the ferrets would track the ball. Outcomes were reported semi-quantitatively with three categories: normal visual response (0), abnormal visual response (1), and no visual response (2).

Ferrets were euthanized at P52, within 24 h of the final MR imaging, using isoflurane anesthesia (5%) followed by exsanguination by transcardiac perfusion with 0.1 M phosphate buffered saline. The brains were excised, weighed, and cut in the parasagittal plane. Samples of dorsal frontal cerebrum and dorsal parietal cerebrum were frozen. The left hemisphere was immersion fixed in cold 10% buffered formalin for 2–3 weeks, sliced in the coronal plane, dehydrated, and embedded in paraffin. Paraffin blocks were sectioned (6 μm thickness) for staining with hematoxylin + eosin and with solochrome cyanin + eosin for examination of myelin. Corpus callosum thickness was measured at the midline and above the lateral angle of the lateral ventricle. Immunostains included: rabbit polyclonal anti-glial fibrillary acidic protein (GFAP; 1:10,000 dilution; DAKO Z0334; Glostrup, Denmark) to label astrocytes; rabbit polyclonal anti-Ki67 (1:250 dilution; Novocastra NCL-Ki67p; Newcastle upon Tyne, UK) to label proliferating cells in the subventricular zone (SVZ); and rabbit polyclonal anti-amyloid-β precursor protein (APP; 1:3000 dilution; Invitrogen 36-6900; Camarillo, CA, USA) to identify damaged axons. GFAP content was measured in frozen frontal lobe samples using ELISA as previously described in detail [8].

Quantitative data (mean ± standard error of the mean) were analyzed to ensure a normal distribution, and *p* values ≤ 0.05 were deemed statistically significant. Statistical analyses were performed for the weight, ventricle size, and behavioral tests at multiple time points. Weights, behavioral test, ventricle size, histological, and ELISA data were analyzed using analysis of variance (ANOVA) and two-tailed *t*-tests (SPSS 19.0 software program). Vision data were analyzed using Chi square. Histological cell counts, corpus callosum thickness, and GFAP ELISA analyses were conducted only on ferrets that completed the 2-week drug trial (n = 17).

## Results

**Fig. 1:**
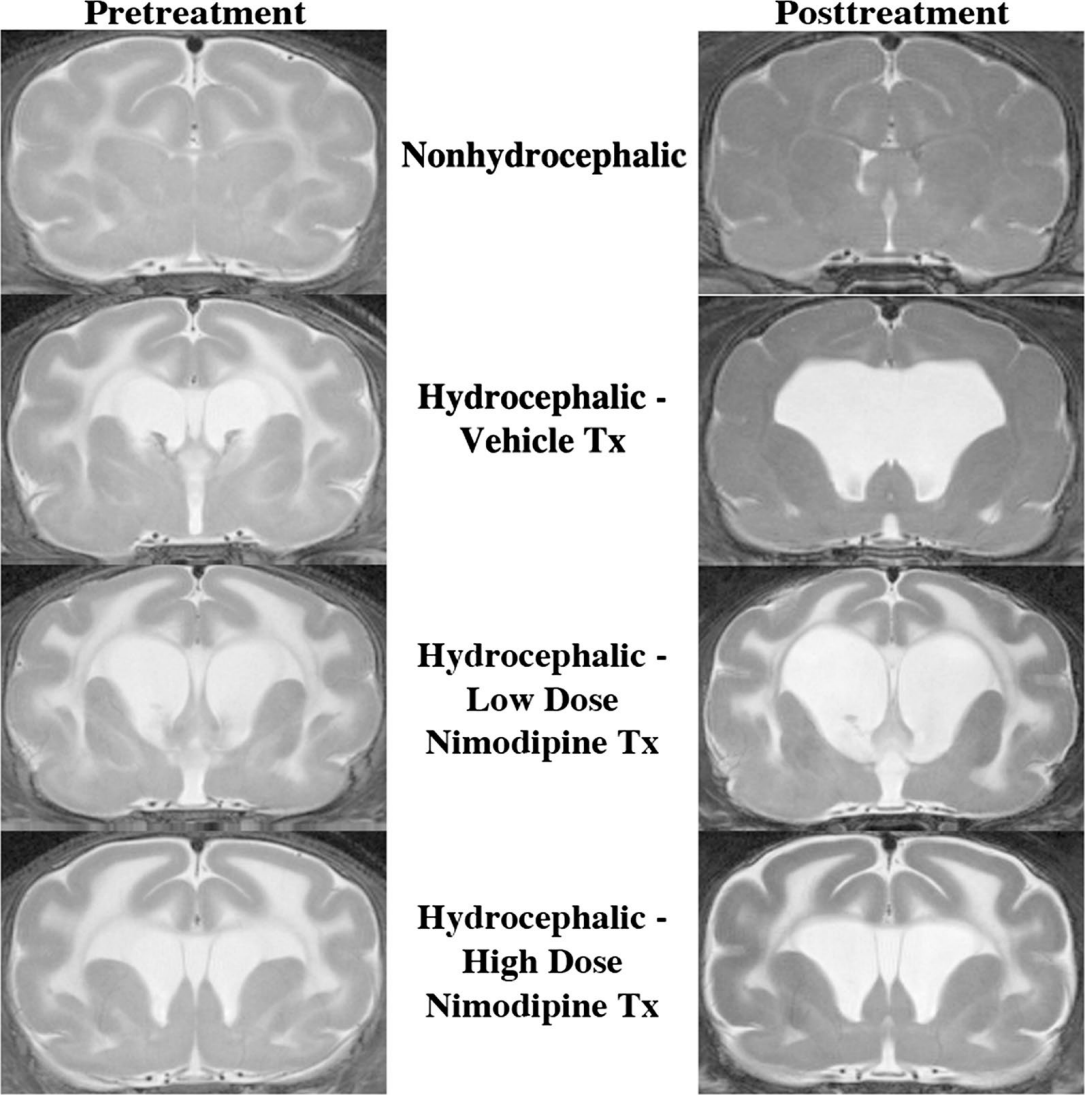
T2-weighted magnetic resonance (MR) images of ferret brains depicting the frontal horns of the lateral ventricles in coronal slices. The pretreatment images were obtained at age 29 days (P29), 2 weeks after kaolin injection into the cisterna magna, and the posttreatment images were obtained at P52, after 2 weeks drug therapy. In non-hydrocephalic ferrets (top row) the ventricles are barely visible at both ages. In hydrocephalic ferrets of all three treatment groups, the ventricles expanded progressively during the treatment period. Increased signal intensity (due to increased water content) in the cerebral white matter was observed in all hydrocephalic treatment groups

Thirty-three ferrets were injected with kaolin and two with saline. Nine died within 2 days of kaolin injection and nine more died as a complication of the initial vehicle solvent formulation. Table 1 shows the initial sample size (“Intent to treat”) and the final sample size after accounting for all mortality. Kaplan–Meier survival analysis performed on hydrocephalic ferrets during the 2-week treatment period showed no significant difference in survival between sham, low dose, and high dose nimodipine-treated groups (*p* = 0.443 to *p* = 0.532). On MR images, mild ventriculomegaly was apparent by 2 days after kaolin injections, particularly involving the lateral ventricles. At 14 days post-kaolin, ventricular expansion was mild to severe (Fig. 1; *p* < 0.005, *t* tests). All three hydrocephalic treatment groups showed further ventricular enlargement of the lateral and fourth ventricles during the treatment period (to P52). However, the enlargement did not differ between groups (*p* > 0.05, ANOVA; Table 1).

**Table 1:** Results of nimodipine treatment in hydrocephalic ferrets

	Non-hydrocephalic controls	Vehicle treated hydrocephalus	Low dose nimodipine hydrocephalus	High dose nimodipine hydrocephalus
Sample size (initial intent to treat)	11	5	5	5
Lateral ventricle area index (P16)	0.037 ± 0.004	0.092 ± 0.022*	0.079 ± 0.012*	0.100 ± 0.023*
Lateral ventricle area index (P29/pre-treatment)	0.023 ± 0.003	0.153 ± 0.042*	0.145 ± 0.037*	0.184 ± 0.046*
Body weight (g) (P29/pre-treat)	136.8 ± 8.0	114.7 ± 9.6	122.3 ± 12.6	120.2 ± 9.9
Survivors after early mortality related to vehicle	7	3	4	3
Lateral ventricle area index (P29-censored after exclusion of early deaths)	0.023 ± 0.004	0.153 ± 0.042*	0.123 ± 0.038*	0.185 ± 0.083*
Lateral ventricle area index (P52/post-treatment)	0.027 ± 0.003	0.192 ± 0.067*	0.238 ± 0.112	0.303 ± 0.122*
Enlargement ventricles during treatment (%)	–	25.2 ± 32.3	93.5 ± 36.0	63.7 ± 27.2
Body weight (g) (P29-censored after exclusion of early deaths)	125.4 ± 8.5	114.7 ± 9.6	120.8 ± 11.2	128.0 ± 9.2
Body weight (g) (P52/post-treat)	299.9 ± 13.7^#^	288.5 ± 42.5^#^	272.7 ± 58.7^#^	310.5 ± 4.5^#^
Body weight increase during treatment (%)	139.2 ± 22.7	151.5 ± 35.5	125.7 ± 43.6	142.6 ± 41.8
Rearing activity (beam breaks per 3 min) (P48–50)	112 ± 15	88 ± 19	29 ± 18*	84 ± 53
Ambulatory activity (beam breaks per 3 min) (P48–50)	700 ± 74	604 ± 88	1143 ± 615	971 ± 225
Total activity (beam breaks per 3 min) (P48–50)	930 ± 68	783 ± 110	1353 ± 670	1153 ± 230
Open field—number cells entered (per 3 min) (P48–50)	122 ± 14	126 ± 12	259 ± 146	129 ± 17
Open field—percent cells entered (per 3 min) (P48–50)	64 ± 6	71 ± 4	51 ± 9	59 ± 10
Open field—distance traveled (m per 3 min) (P48–50)	7.83 ± 0.96	7.77 ± 0.79	18.27 ± 12.25	7.84 ± 1.21
Diminished vision/blind (P48–50)	0/0	0/1	0/0	1/2
Medial corpus callosum thickness (μm)	544 ± 44	288 ± 74*	198 ± 29*	291 ± 79*
Lateral corpus callosum thickness (μm)	397 ± 32	272 ± 100	204 ± 23*	269 ± 73
Ki-67 positive cells ratio in SVZ (%)	16.1 ± 2.4	7.0 ± 1.9*	15.5 ± 3.5	7.1 ± 2.3*
GFAP content (ELISA) frontal cerebrum (μg GFAP/g protein)	0.446 ± 0.088	0.640 ± 0.361	0.373 ± 0.037	0.572 ± 0.178

Data are presented as mean ± standard error of the mean (SEM)

Behavior and ventricle size are specified at by postnatal day (P) age in days. Histological and glial fibrillary acidic protein (GFAP) data are at P52

*p < 0.05 control vs. hydrocephalic, t tests or ANOVA

^#^p < 0.05 P29 (pre-treat) vs. P52 (post-treat), t tests

Hydrocephalic and non-hydrocephalic ferrets reached motor and behavioral developmental milestones at approximately the same time. All were crawling by P27–P29 and started walking between P34 and 37 [8]. Hydrocephalic ferrets tended to display higher activity scores during the first week after kaolin injections. During the second week after kaolin (P24–26), non-hydrocephalic ferrets showed more ambulation and activity than hydrocephalic ferrets (*p* < 0.010). There were no consistent behavioral differences between the three hydrocephalic groups during the drug trial phase (Table 1). Four hydrocephalic ferrets exhibited severe visual impairments during the last week of the study (Table 1); they tended to circle continuously in their enclosures.

**Fig. 2:**
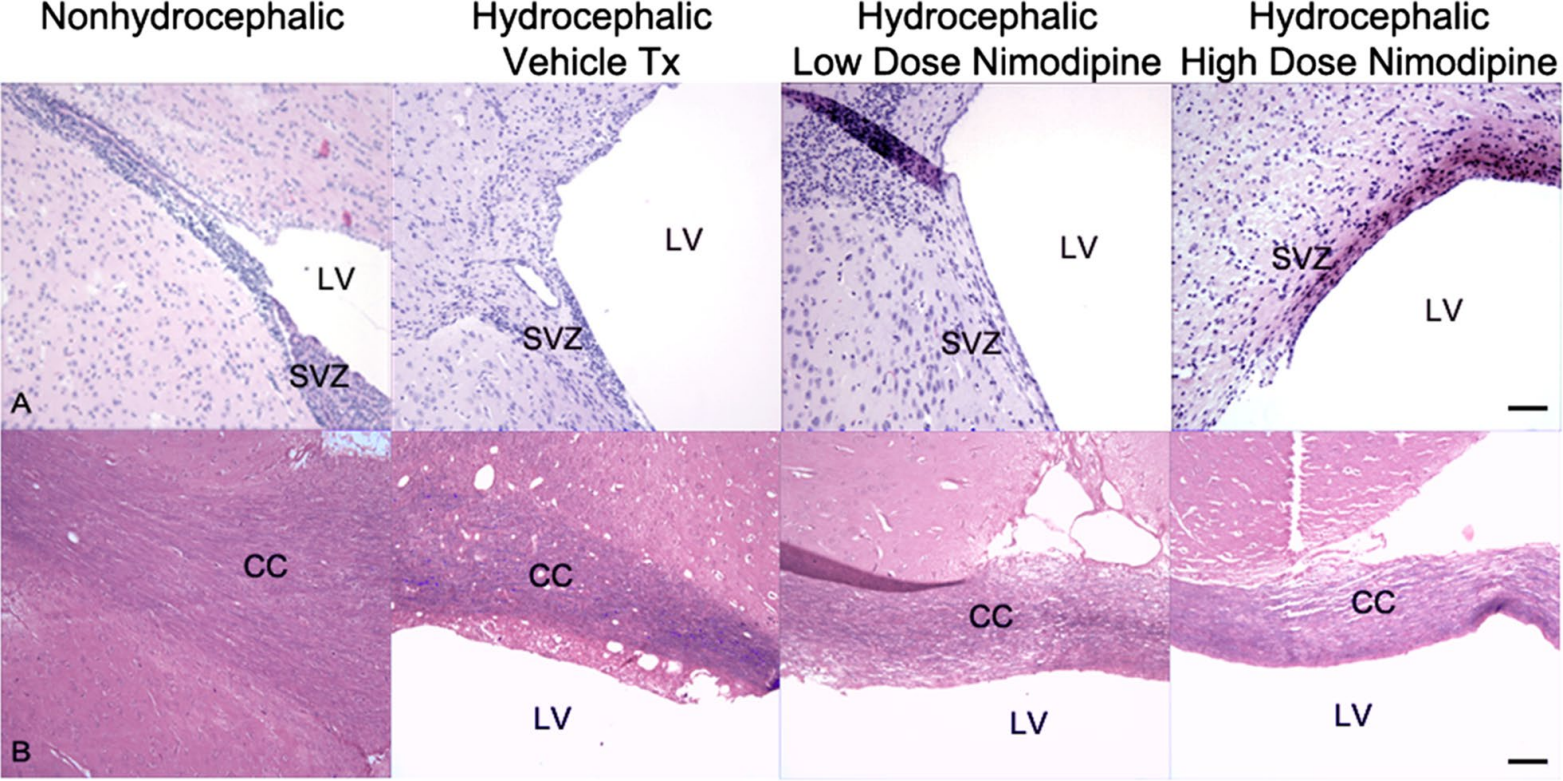
Photomicrographs showing periventricular brain tissue after sacrifice (at age 52 days) of non-hydrocephalic and hydrocephalic ferret brains following a treatment period that lasted 14 days. Upper row (**A**) shows the dorsolateral angle of the frontal horn of the lateral ventricle (LV) (hematoxylin and eosin stain). In normal animals, the subventricular zone (SVZ) is densely cellular whereas in hydrocephalic animals from all groups the SVZ was less pronounced. Lower row (**B**) shows the roof of the frontal horn including the corpus callosum (CC) (solochrome cyanin stains myelin blue, with eosin counterstain pink). In all hydrocephalic treatment groups, the CC was thin (see Table 1 for quantitative data) and the periventricular white matter rarified or frayed with less intense myelin staining. Total magnification ×100; scale bar = 100 μm

Histologic evaluation of the hydrocephalic ferrets showed that ventriculomegaly was accompanied by compression of periventricular white matter (Fig. 2), focal fraying of the corpus callosum, and reduced cellularity at the dorsolateral angle of the frontal horn. Where white matter was intact, the intensity of myelin staining was similar in hydrocephalic and non-hydrocephalic ferrets. However, damaged periventricular white matter regions displayed reduced myelin staining (Fig. 2). The corpus callosum was thinner in hydrocephalic animals than in non-hydrocephalic animals (*p* = 0.005, *t* test), but there were no statistically significant differences between the hydrocephalic treatment groups (Table 1). In the severely hydrocephalic ferrets, particularly those with visual impairment, damaged axons with APP immunoreactivity were abundant in the parietal-occipital white matter (not shown).

**Fig. 3:**
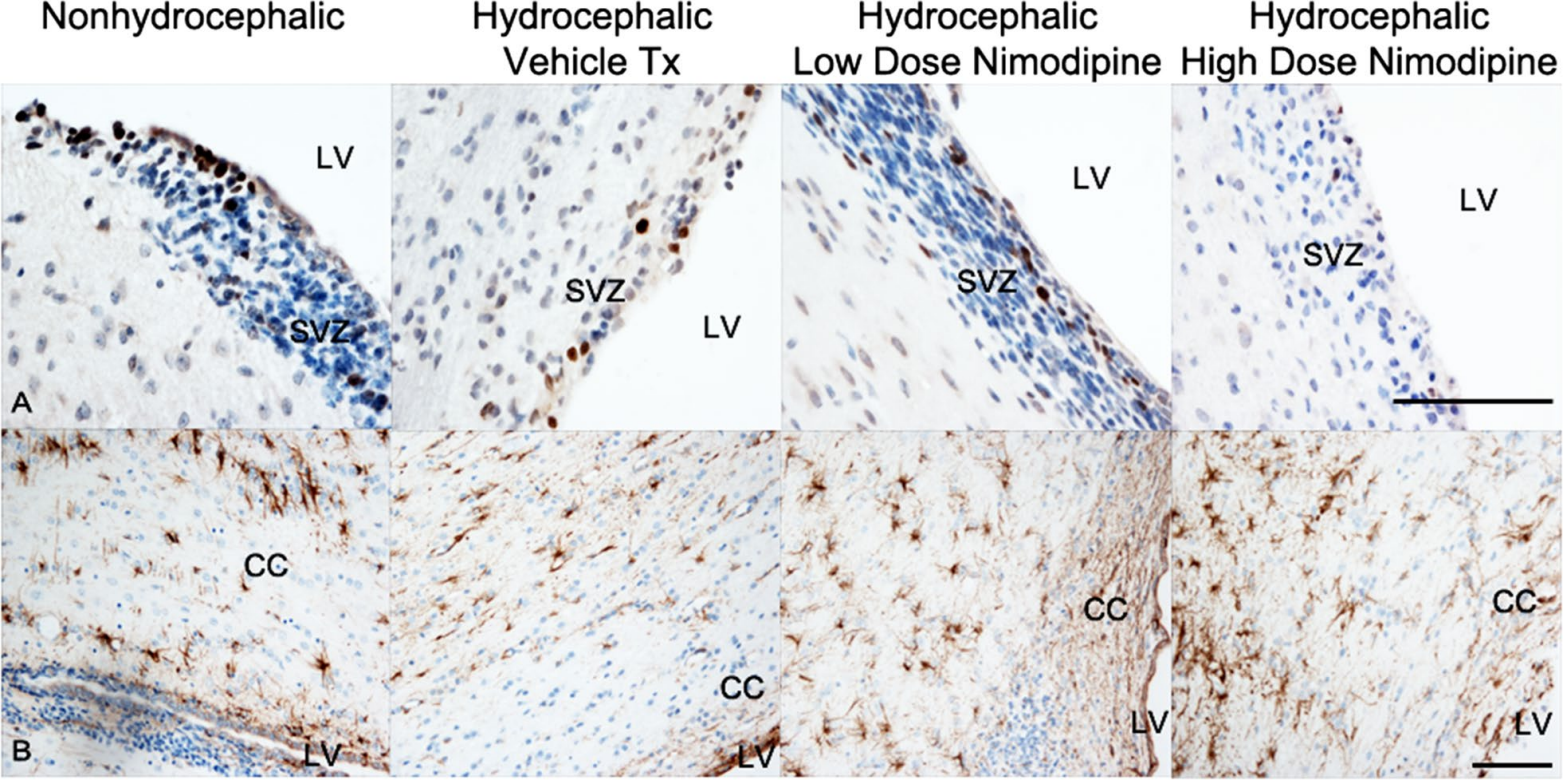
Photomicrographs showing periventricular brain tissue after sacrifice (at age 52 days) of non-hydrocephalic and hydrocephalic ferret brains following a treatment period that lasted 14 days. Upper row (**A**) shows Ki67 immunostaining (positive nuclei of proliferating cells brown; with hematoxylin counterstain) at the dorsolateral angle of the frontal horn of the lateral ventricle (LV). In normal animals, the subventricular zone (SVZ) contains many proliferating cells whereas labeled cells are fewer in the hydrocephalic animals (see Table 1 for quantitative data). Lower row (**B**) shows GFAP immunostaining (positive astrocytes brown; with hematoxylin counterstain) in the corpus callosum (CC) above the LV. Astroglial hypertrophy is subtle and there are no obvious differences between the three hydrocephalic ferret treatment groups. Total magnification **A**—×400, **B**—×200; Scale bar = 100 μm

Non-hydrocephalic ferrets have proliferating cells demonstrable by Ki67 immunoreactivity in the residual SVZ at the dorsolateral angle of the frontal horn (Fig. 3). Untreated and high dose nimodipine-treated hydrocephalic ferrets had a thinner SVZ and fewer Ki67 positive cells compared to controls (Table 1). Hydrocephalic ferrets had GFAP-immunoreactive hypertrophic reactive astrocytes in the white matter; there were no obvious differences between the hydrocephalic drug treatment groups (Fig. 3). ELISA of frontal lobe homogenates showed elevated GFAP content in untreated and high dose nimodipine hydrocephalic ferrets compared to non-hydrocephalic controls, but the difference was not significant (Table 1; *p* = 0.676, *t* test).

## Discussion

Nimodipine was previously shown to have mild neuroprotective effects when administered for 2 weeks to juvenile rats with kaolin induced experimental hydrocephalus [7]. However, this study revealed that comparable doses in young hydrocephalic ferrets did not offer any consistent behavioral, histological, or biochemical improvements. Although there was normalization of GFAP content and Ki67 counts in ferrets treated with low dose nimodipine, that group had the greatest expansion of the ventricles during the drug administration phase.

Why did the nimodipine treatment, as well as a magnesium sulfate treatment [9], fail in ferrets when they had been modestly protective in hydrocephalic rats? It could be a species-specific matter or possibly related to the degree of brain maturation. Hydrocephalic rats were treated from 5 to 7 weeks age, which in relative terms of cerebral myelination is slightly more mature than the ferrets studied here (http://translatingtime.org/ [10]) [8, 11]. There is also a possibility that the treatment was applied late to mitigate severe damage; we had too few cases to correlate ventricle size with magnitude of treatment effect.

It is also possible that the mode of drug delivery played a role. Hydrocephalic rats received a continuous infusion of nimodipine via osmotic minipumps. In this case there was a concern about maternal rejection [12], therefore we applied the drug by subcutaneous injections. In dogs (which along with ferrets are of the Caniformia suborder within the order Carnivora) the elimination half-life of nimodipine after intravenous injection and after ~ 5 mg/kg oral dose is ~ 1.8 h [13]. Therefore, it is likely that the ferrets’ brains were not exposed continuously to nimodipine. An implantable delivery system for nimodipine treatment of subarachnoid hemorrhage has been developed [14], but it is prohibitively expensive. Sustained release oral preparations are also under development [15].

The most surprising complication in this experiment was initial mortality attributable to the vehicle mixture of DMSO and ethanol, which was identical to that used in our rat experiment [7]. DMSO is generally considered to be safe [16]. However, chronic ocular toxicity is greater in dogs than in rats [17] and it is likely that metabolism of DMSO differs between very young ferrets and adults [18].

In summary, 2-week intermittent subcutaneous dosing of nimodipine to hydrocephalic ferrets from age 38 to 52 days was not shown to be beneficial. Timing, administration route, and dosage may have all contributed to the ineffective therapeutic outcome. While we do not absolutely reject the proposal that nimodipine can modulate damage in immature brains with hydrocephalus, this experiment shows that the window of opportunity might be narrow. More robust preclinical studies with continuous delivery or administration of long-acting nimodipine are required before considering a clinical trial in humans.

## Abbreviations

ANOVA: analysis of variance
CBF: cerebral blood flow
CC: corpus callosum
CSF: cerebrospinal fluid
DMSO: dimethyl sulfoxide
ELISA: enzyme linked immunosorbent assay
GFAP: glial fibrillary acidic protein
MR: magnetic resonance
SVZ: subventricular zone
